# Antidepressant prescription practice and related factors in Switzerland: a cross-sectional analysis of health claims data

**DOI:** 10.1186/s12888-019-2178-4

**Published:** 2019-06-24

**Authors:** Elisa Haller, Birgit Watzke, Eva Blozik, Thomas Rosemann, Oliver Reich, Carola A. Huber, Markus Wolf

**Affiliations:** 1 Department of Psychology, University of Zurich, Binzmühlestrasse 14/16, Zurich, Switzerland; 2 Department of Health Sciences, Helsana, Zurich, Switzerland; 3 Department of Primary Care, University of Zurich, Zurich, Switzerland

**Keywords:** Antidepressant prescription, Mental health care, Depression, Regional variation

## Abstract

**Background:**

The aim of the study was to examine the prevalence of and factors associated with antidepressant (AD) prescriptions in order to draw a comprehensive picture of prescribing practices in Switzerland.

**Method:**

We conducted a population-based, cross-sectional descriptive study using a large Swiss healthcare claims database, covering approximately 13% of the Swiss population. AD prescription was determined by identifying patients (*N* = 105,663) with health claims data of at least 1 AD prescription in the year 2016. AD medication was identified using ATC-codes classified by the World Health Organisation. Univariate, bivariate and multivariate analyses using logistic regression were performed.

**Results:**

The extrapolated 1-year prevalence of AD prescription was 8.7% (95% CI, 8.7–8.8) with two thirds of AD recipients being female and the average age being 59 years (SD = 19.1). The regional distribution of prescription rates varied between cantons and ranged from 6.5 to 11.7%. Logistic regression revealed higher prescription rates among females compared to males (OR: 1.52) and an increased probability of AD prescription by age up until 54 years (OR: 2.25) and ≥ 85 years (OR: 2.32). Comorbidity is associated with higher odds (OR: 3.26 with 1–2 comorbidities) and enrollment in a managed care plan (compared to standard care) with lower odds for an AD prescription (OR: 0.85).

**Conclusion:**

This study is the first in Switzerland to describe the prevalence of and factors associated with AD prescription based on a large health claims database reflecting routine care. The results provide important information about regional variation, prescription source, and potential over-prescription in the treatment of depressive disorders.

**Electronic supplementary material:**

The online version of this article (10.1186/s12888-019-2178-4) contains supplementary material, which is available to authorized users.

## Background

Antidepressants (ADs) are the most commonly prescribed class of psychotropic drugs with properties of reducing symptoms of low mood and motivation [1]. They are predominantly used for the treatment of depressive disorders, a highly prevalent condition, associated with high economic burden and personal suffering [2]. Besides psychotherapeutic approaches, AD medication is an evidence-based treatment that is recommended by international guidelines for moderate to severe depression as well as for chronic courses of the disorder (e.g. [3, 4]). The use of AD for mild forms of depression is not a first line treatment but can be considered after a careful weighing of benefits and risk, most notably adverse side effects. Therefore, guidelines for depression differentiate their recommendations for AD use according to severity and form of depression.

The prescription rates of ADs have increased substantially over the last few decades worldwide. In the National Health and Nutrition Examination Surveys (2005–2008) 11% of all Americans aged 12 and older reported having taken an AD within the past month, indicating an increase in prescription rate by 400% since the time period 1988 to 1994 [5]. The number of AD prescriptions issued in England has more than doubled over a period of 13 years, according to analyses based on the Prescription Cost Analysis Survey [6]. Similar trends are observed in many other industrialised countries (e.g., [7, 8]) and also in patients with somatic conditions, such as coronary heart disease, which involve the danger of potentially adverse interactions [9]. Attempts to explain the increase in AD prescriptions and AD use have raised concerns about inappropriate health care. There is evidence that the increase in AD prescription is driven by long-term use of AD [10], but researchers also argue that the widespread use is likely due to extension of indications for AD treatment [11]. Another underlying factor for the increase in AD use is suspected to be overprescription in less severe forms of depression. This aspect is particularly relevant given that a large volume of patients with mild to moderate depression are treated in primary care, where psychopharmacological medication is the most commonly applied form of treatment [12]. Moreover, the rise in AD prescription has also been linked to the introduction and use of new AD classes, such as selective serotonin reuptake inhibitors (SSRIs) (e.g., [10]) and serotonin-norepinephrine reuptake inhibitors (SNRIs), which are associated with better tolerability. These findings give reason to assess the class of ADs being prescribed and the type of provider that prescribes AD medication.

Reliable and representative data for the outpatient treatment of depressed patients is fragmentary but preliminary data indicates that the Swiss population is treated mainly with medication [13]. One population-based source of information about levels of distress and health service use is the Swiss national health survey (Swiss Health Survey (SHS) [14]). The survey is conducted via telephone interviews and a subsequent questionnaire in a five-year cycle. While the SHS provides a valuable data source for the health status, health behaviour and health services use in the Swiss population, information are based on self-reports and refers to the utilisation of an array of drugs and health services. The assessment method used might lack precision about the specific type and source of the prescription and carries the risk of inaccurate recalling and reporting. This type of information needs to be complemented with objective and fine-grained data on psychopharmacological drug use. In summary, data availability on AD dispensing can be considered incomplete [14]. The Swiss healthcare system is organised in a decentralised manner with the possibility of accessing primary and secondary care free of choice. A central registry for medication dispensing does not exist. Routine data based on insurance claims provide a unique source of information in order to increase our understanding of AD prescription practices.

The objective of the current study was 1) to estimate the AD prescription prevalence in Switzerland including regional variations, 2) to determine factors associated with AD use, and 3) to quantify the prescription rate by AD classes and type of health care provider.

## Methods

### Study design and study population

The present study used a cross-sectional and population-based approach. The analysis was based on routine data from the Helsana group, a large health insurer covering approximately 13% of the Swiss population. Health insurance is obligatory in Switzerland, which means that all Swiss residents are required to contract basic health insurance on the private market. Every insured person chooses a monthly premium and a deductible ranging from Swiss Francs (CHF) 300 to 2500 per year. Health care plans comprise either standard care models or managed care models, including family doctor and telemedicine models. Health insurance claims data were used from all patients insured with Helsana in 2016, a collective of ca. 1.17 million individuals. The recorded insurance claims cover almost all health care and pharmacy invoices, including costs of health care use and prescription drugs. Data are approximately representative of the Swiss population. According to the Swiss national ethical and legal regulations, ethical approval from the local ethics committee (ethical committee of the canton Zurich) was not needed for the study.

### Identification of patients with AD use

For the purpose of the current analysis all insurees who were prescribed at least 1 AD in 2016 were included in the analysis. AD medication was grouped according to the Anatomical Therapeutic Chemical (ATC) classification system of the World Health Organisation [15]. This system categorises drugs according to their therapeutic and chemical characteristics. ADs were classified under N06Ax, with the fourth level indicating the class of AD as follows: 1) tricyclic ADs (TCAs, code N06AA), 2) selective serotonin reuptake inhibitors (SSRIs, code N06AB), 3) monoamine oxidase inhibitors (MAOIs, codes N06AF + N06AG), and 4) other ADs (N06AX), which subsume nonuniform underlying mechanisms of action (atypical AD), such as serotonin-norepinephrine reuptake inhibitors (SNRIs) and tetracyclic ADs [15].

### Population and prescriber characteristics

Population characteristics included gender, age groups, regional variables such as language and area of residence, health insurance plan (managed care or standard plan) and type of deductible class (low: ≤ CHF 500, high: > CHF 500), and amount and type of comorbid chronic conditions (CCCs).

Regional variables included the greater regions of Switzerland, which are reference areas defined by the Swiss Federal Office of Statistics (Zurich, Espace Mittelland, Lemanic region, Northwestern Switzerland, Eastern Switzerland, Ticino, and Central Switzerland) and the 26 member states (cantons) of the Swiss confederation at canton-level. The area of residence displayed the level of urbanisation and was classified into the categories “urban” and “rural”, according to the national community typology of the Swiss Federal Office of Statistics [16]. Urban and intermediary (urban area) included (1) municipalities of small, medium-large and large agglomerations or outside an agglomeration, and (2) dense urban areas and rural centres (periurban municipalities with medium and high density, rural centre municipalities). Rural areas were defined as (1) low-density periurban communities, and (2) rural, central and peripheral communities.

A total of 21 CCCs were identified using a proxy variable for diagnoses developed on the basis of medication prescriptions related to chronic conditions [17]. For each CCC, the point-prevalence was calculated by dividing the number of persons with at least one medication prescription in one of the defined ATC-groups by the total of patients within the given year.

Characteristics of the prescription patterns comprise the prescription source (type of health care provider) and the class of ADs. In Switzerland, physicians are the only legal health care provider, who are allowed to prescribe drugs. Defined by their practice speciality, the following provider categories were grouped: general practitioner (GP) only, psychiatrist only, other medical specialist only (e.g., gastroenterologist), hospital ambulatory only and combination of providers. The combination category refers to all people, who received their AD prescription from two different types of providers (e.g., GP and psychiatrist or another specialist; or psychiatrist and another specialist, etc.). This is the case for individuals who received more than one prescription from at least two different providers.

### Statistical analyses

Frequencies and proportions are reported in absolute numbers and percentages of the sample. Chi-square-tests and Wilcoxon rank-sum tests were used to compare the proportions of baseline characteristics and the presence of CCCs in persons with and without AD prescription. Chi-square-tests were used to test the association between the type of provider of AD prescriptions and the area of residence of AD recipients. Prevalence rates are provided both raw and extrapolated to the entire Swiss population using census data from the Swiss Federal Office of Statistics. The direct comparison between the raw and the adjusted results allows us to test generalizability of our data to the entire Swiss population. The procedure of extrapolation was used to adjust for age, gender and canton of residence. Multivariable logistic regression was employed to examine factors associated with AD prescription (dependent variable). Independent variables included in the regression model were age groups (15–24 (reference group), 35–44, 45–54, 55–64, 65–74, 75–84, ≥85 years), gender (female or male), insurance plan (managed care or standard plan), type of deductible class (low or high deductible), residence area (rural or urban), and number of comorbid conditions. Estimates are presented as odds ratios (ORs) with 95% confidence interval (CIs). All analyses were performed using R version 3.2.0 (R Development Core Team 2015). A *p*-value < 0.05 was considered statistically significant.

## Results

### Prescription prevalence and description of the study population

Our procedure identified 105,663 persons (male: 6.2%, female: 11.7%) who received one or more AD prescriptions in 2016, representing a 1-year AD prescription prevalence of 9.0% (95%-CI: 9.0–9.1%) in the study population covering more than 1.17 million health insurance members. The extrapolated overall prevalence is 8.7% (95%-CI: 8.7–8.8%) and remains 6.2% for men and 11.7% for women after extrapolating to the general Swiss population. Characteristics of the study population and the groups with and without AD prescriptions are presented in Table 1.

**Table 1:** Characteristics of the study population

	Total N (%)	With AD use n (%)	Without AD use n (%)
Total	1,169,489 (100)	105,663 (100)	1,063,826 (100)
Gender			
Male	567,102 (48.5)	34,927 (33.1)	532,175 (50.0)
Female	602,387 (51.5)	70,736 (66.9)	531,651 (50.0)
Age yrs. Mean (SD)	43.4 (24.4)	59.3 (19.1)	41.8 (24.3)
Age group (yrs.)			
0–14	180,099 (15.4)	178 (0.2)	179,912 (16.9)
15–24	125,114 (10.7)	3895 (3.7)	121,219 (11.4)
25–34	140,438 (12.0)	7872 (7.5)	132,566 (12.5)
35–44	152,784 (13.1)	12,914 (12.2)	139,870 (13.1)
45–54	160,529 (13.7)	18,741 (17.7)	141,788 (13.3)
55–64	142,547 (12.2)	18,696 (17.7)	123,851 (11.6)
65–74	128,998 (11.0)	16,481 (15.6)	112,517 (10.6)
75–84	92,173 (7.9)	15,957 (15.1)	76,216 (7.2)
≥ 85	46,807 (4.0)	10,929 (10.3)	35,878 (3.4)
Region			
Zurich	278,505 (23.8)	24,481 (23.2)	254,024 (23.9)
Espace Mittelland	224,446 (19.2)	21,213 (20.1)	203,233 (19.1)
Lemanic region	188,171 (16.1)	17,208 (16.3)	170,963 (16.1)
Northwestern Switzerland	160,304 (13.7)	13,976 (13.2)	146,328 (13.8)
Eastern Switzerland	145,167 (12.4)	13,425 (12.7)	131,742 (12.4)
Ticino	71,389 (6.1)	7786 (7.4)	63,603 (6.0)
Central Switzerland	101,507 (12.4)	7574 (7.2)	93,933 (8.8)
Area of residence			
Urban area	912,328 (78.0)	83,854 (79.4)	828,474 (77.9)
Rural area	257,161 (22.0)	21,809 (20.6)	235,352 (22.1)
Managed Care			
Yes	737,434 (63.1)	54,472 (51.6)	682,962 (64.2)
No	432,055 (36.9)	51,191 (48.4)	380,864 (35.8)
Franchise			
High	308,618 (26.4)	10,351 (9.8)	298,267 (28.0)
Low	860,871 (73.6)	95,312 (90.2)	765,559 (72.0)

*AD* Antidepressant; For the definition of the variables area of residence (urban vs. rural), managed care (Yes, No), and Franchise (High vs. Low): see Methods section

Two thirds of all AD recipients were female, and the average age of the sample was 59 years (SD = 19.1). Of all AD users, 11.2% were adolescents or young adults (age 15–34) whereas 41% of the AD receivers are aged ≥65 years. The prescription prevalence in age group ≥65 years was 16.2% (data not shown). Almost half of the female AD users were ≥ 65 years old (44%) compared to 35% of the male AD users aged ≥65 years. Extrapolated and unadjusted prevalence rates did not differ in age-related subgroups (Additional file 1: Figure S1). Approximately half of the AD recipients were part of the managed care plan (51.6%), whereas almost two thirds of persons without AD prescriptions (64.2%) were insured with this type of plan. Overall, comorbid diseases were more prevalent in the patient group receiving AD prescriptions compared to the group of patients without AD prescriptions (Table 2). For instance, 50.1% of the patients with AD use also received treatment for cardiovascular diseases compared to approximately 21.3% of patients without AD use. Other conditions, which were more prevalent in AD recipients than in persons without AD prescription were acid-related disorders (47.6% vs. 17.0%), epilepsy, psychoses, pain, iron deficiency anaemia, Parkinson’s disease and osteoporosis.

**Table 2:** Type and prevalence of comorbid chronic conditions in the study population with and without AD use

	Total N (%)	With AD use N (%)	Without AD use N (%)	χ^2^ (df)	*P*
Chronic conditions					
Acid related disorders	231,616 (19.8)	50,245 (47.6)	181,371 (17.0)	56,306 (1)	< 0.001^a^
Bone diseases (osteoporosis)	21,143 (1.8)	5007 (4.7)	16,136 (1.5)	5618.5 (1)	< 0.001^a^
Cancer	15,955 (1.4)	3076 (2.9)	12,879 (1.2)	2064.2 (1)	< 0.001^a^
Cardiovascular diseases (incl. hypertension)	279,956 (23.9)	52,975 (50.1)	226,981 (21.3)	43,782 (1)	< 0.001^a^
Dementia	20,062 (1.7)	6808 (6.4)	13,254 (1.2)	15,395 (1)	< 0.001^a^
Diabetes mellitus	56,381 (4.8)	10,877 (10.3)	45,504 (4.3)	7581.5 (1)	< 0.001^a^
Epilepsy	33,742 (2.9)	13,474 (12.8)	20,268 (1.9)	40,354 (1)	< 0.001^a^
Glaucoma	38,163 (3.3)	6719 (6.4)	31,444 (3.0)	3525.2 (1)	< 0.001^a^
Gout, Hyperuricemia	18,380 (1.6)	2818 (2.7)	15,562 (1.5)	900.13 (1)	< 0.001^a^
HIV	2365 (0.2)	417 (0.4)	1948 (0.2)	212.07 (1)	< 0.001^a^
Hyperlipidemia	125,564 (10.7)	23,948 (22.7)	101,616 (9.6)	17,242 (1)	< 0.001^a^
Intestinal inflammatory diseases	5144 (0.4)	1135 (1.1)	4009 (0.4)	1065.7 (1)	< 0.001^a^
Iron deficiency anemia	56,746 (4.9)	10,527 (10.0)	46,219 (4.3)	6570.1 (1)	< 0.001^a^
Migraines	12,958 (1.1)	3252 (3.1)	9706 (0.9)	4110.9 (1)	< 0.001^a^
Pain	106,865 (9.1)	29,862 (28.3)	77,003 (7.2)	51,163 (1)	< 0.001^a^
Parkinson’s disease	10,846 (0.9)	4233 (4.0)	6613 (0.6)	11,979 (1)	< 0.001^a^
Psychoses	35,061 (3.0)	19,466 (18.4)	15,595 (1.5)	95,027 (1)	< 0.001^a^
Respiratory illness (asthma, COPD)	99,180 (8.5)	16,164 (15.3)	83,016 (7.8)	6954.1 (1)	< 0.001^a^
Rheumatologic conditions	138,197 (11.8)	29,732 (28.1)	108,465 (10.2)	29,694 (1)	< 0.001^a^
Thyroid disorders	44,201 (3.8)	10,161 (9.6)	34,040 (3.2)	10,880 (1)	< 0.001^a^
Tuberculosis	883 (0.1)	169 (0.2)	714 (0.1)	108.55 (1)	< 0.001^a^

*CCCs* Classification of comorbid chronic conditions is based on medication prescriptions related to the disorder; ^a^Chi-square test

### Regional variations

**Fig. 1:**
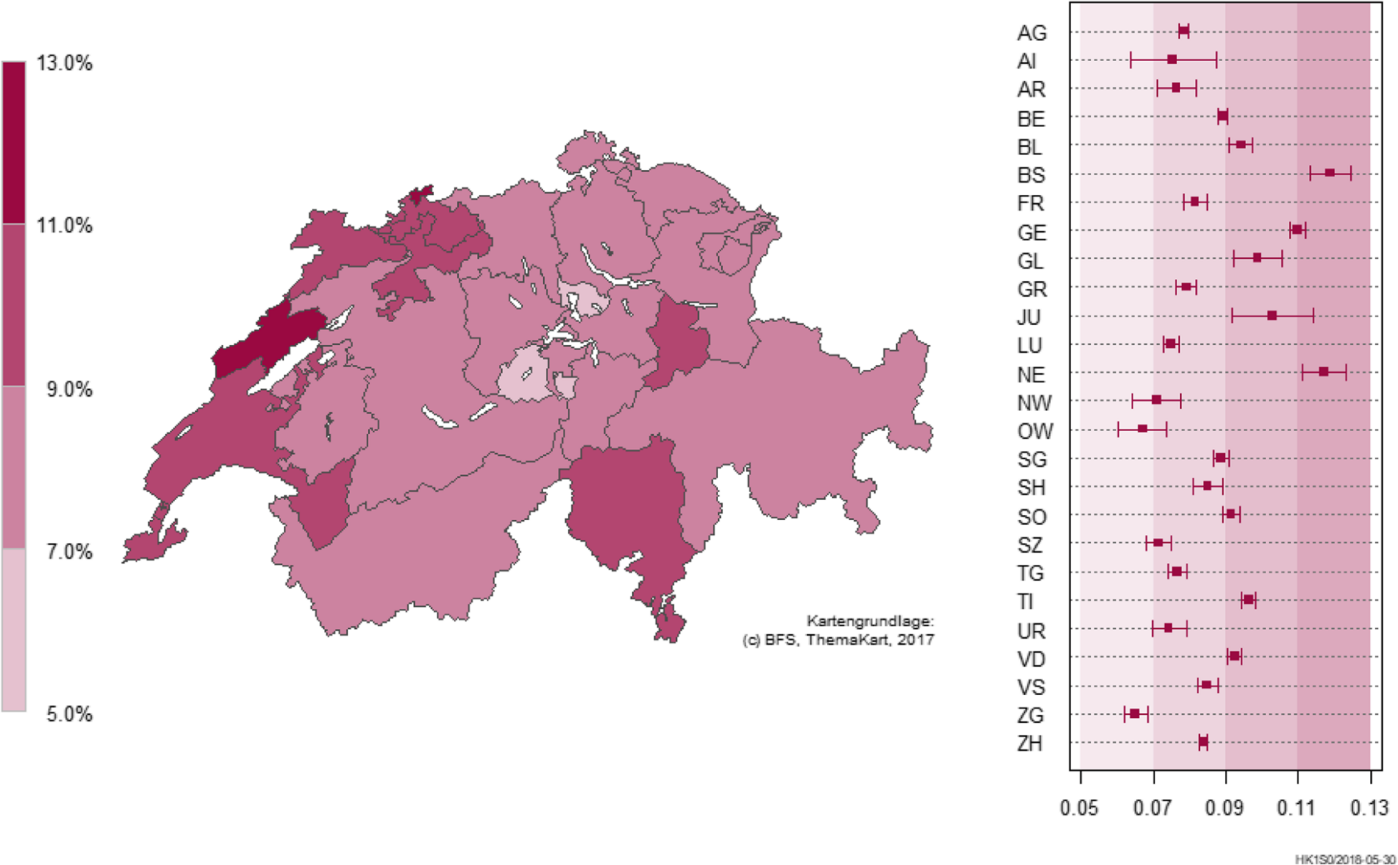
Adjusted AD prescription rates by Swiss cantons in the year 2016. Copyright geodata Swiss Federal Statistical Office / swisstopo

The regional distribution shows that the proportions of individuals using AD were similar across the greater regions of Switzerland in the study population and generally reflect the overall prescription rate (Ticino: 10.9%; see Table 1). However, on a canton-level, there were significant differences in the prevalence rates (Fig. 1), with the highest prevalence in Basel-Stadt (11.9%) and the lowest in Zug (6.5%) when extrapolating to the regional distribution of the Swiss population. The unadjusted and extrapolated prevalence rates differed in some cantons and can be found in the electronic (Additional file 2: Figure S2).

### Factors associated with AD prescription

The results of the multivariable logistic regression are displayed in Table 3. The odds of receiving an AD were 1.52 times higher for women than for men (OR: 1.52, 95%-CI: 1.50–1.54), and AD prescription increased with age until 54 years, with the highest likelihood of AD prescription for people ≥85 years (OR: 2.32, 95%-CI: 2.24–2.41). The odds for AD prescription were smaller in patients covered with managed care compared to patients covered with the standard health insurance plan (OR: 0.85, 95%-CI: 0.84–0.86). The majority of both AD recipients and people without AD prescription (90 and 72%, see Table 1) set a high deductible. Patients with a high deductible were less likely to receive ADs compared to those with a low deductible (OR: 0.44, 95%-CI: 0.43–0.45). Receiving AD prescription was significantly associated with the level of urbanization (lower for patients living in rural than in urban areas (OR: 0.94, 95%-CI: 0.93–0.96). The odds of having an AD prescription increased with the number of comorbid diseases and was 3.25 times higher for persons with 1–2 comorbid conditions (OR: 3.26, 95%-CI: 3.20–3.32) and 13.79 times higher for patients showing ≥5 comorbid conditions (OR: 13.79, 95%-CI: 13.45–14.14) compared to AD users without such a condition.

**Table 3:** Logistic regression analysis for predictors of AD prescription

Variables	AD prescription	
	OR (95% CI)	*P*
Age group (yrs.)^a^		
0–14	0.03 (0.03–0.03)	< 0.001
15–24	1.0	
25–34	1.50 (1.45–1.55)	< 0.001
35–44	2.03 (1.97–2.10)	< 0.001
45–54	2.25 (2.18–2.32)	< 0.001
55–64	1.81 (1.76–1.87)	< 0.001
65–74	1.34 (1.29–1.38)	< 0.001
75–84	1.67 (1.61–1.72)	< 0.001
≥ 85	2.32 (2.24–2.41)	< 0.001
Sex (female)	1.52 (1.50–1.54)	< 0.001
Insurance plan (managed care)^b^	0.85 (0.84–0.86)	< 0.001
High deductible^c^	0.44 (0.45–0.43)	< 0.001
Area of residence (rural)^d^	0.94 (0.93–0.96)	< 0.001
Number of Comorbidities^e^		
1–2	3.26 (3.20–3.32)	< 0.001
3–4	6.68 (6.54–6.83)	< 0.001
≥ 5	13.79 (13.45–14.14)	< 0.001

*OR* Odds Ratio, *CI* Confidence Interval; ^a^age group 15–24 is the reference group; ^b^managed care is the reference group; ^c^ high deductible is the reference group; ^d^rural area is the reference group; ^e^No comorbidities is the reference group

### Class and amounts of ADs prescribed

Number and frequencies of individuals with prescriptions by AD class and sociodemographic variables are displayed in Table 4. The most commonly prescribed drug classes were SSRIs (52.3%) and other ADs (51.1%) followed by TCAs (13%) (Table 4). The most commonly prescribed other ADs were: Mirtazapine (15.6%; N06AX11 = atypical AD), Trazodone (11.3%; N06AX05 = atypical AD), Venlafaxine (9.7%; N06AX16 = SNRI), and Duloxetin (7.9%; N06AX21 = SNRI) (data not shown). Another commonly prescribed AD from the class “other AD” was St. John’s wort (14.6%; N06AX25), a herbal medicinal product.

**Table 4:** Number of persons with prescription by AD class and sociodemographics

	SSRIs n (%)	TCA n (%)	MAOIs n (%)	Others n (%)
Total	55,236 (52.3^a^)	13,708 (13.0^a^)	255 (0.2^a^)	53,971 (51.1^a^)
Gender				
Female	37,864 (68.5)	9411 (68.7)	145 (43.1)	35,222 (65.3)
Male	17,372 (31.5)	4297 (31.3)	110 (56.9)	18,749 (34.7)
Age group				
0–14	103 (0.2)	5 (0.1)	0 (0.0)	78 (0.1)
15–24	2284 (4.1)	309 (2.3)	5 (2.0)	1875 (3.5)
25–34	4469 (8.1)	738 (5.4)	20 (7.8)	4033 (7.5)
35–44	7205 (13.0)	1358 (9.9)	41 (16.1)	6701 (12.4)
45–54	9751 (17.7)	2371 (17.3)	51 (20.0)	10,026 (18.6)
55–64	9357 (16.9)	2842 (20.7)	43 (16.9)	9043 (17.9)
65–74	8216 (14.9)	2672 (19.5)	52 (20.4)	7958 (14.7)
75–84	8044 (14.6)	2264 (16.5)	25 (9.8)	8125 (15.1)
≥ 85	5807 (10.5)	1149 (8.4)	18 (7.1)	5522 (10.2)
Area of residence				
Urban area	44,086 (79.8)	10,776 (78.6)	205 (80.4)	42,790 (79.3)
Rural area	11,150 (20.2)	2932 (21.4)	50 (19.6)	11,181 (20.7)

*SSRIs* selective serotonin reuptake inhibitors, *TCA* tricyclic antidepressants, *MAOIs* monoamine oxidase inhibitors; ^a^percentages are related to the subsample with AD prescriptions (*n* = 105,663); all other percentages as related to the total amount per AD class

With regard to gender differences in prescribed AD class, it was apparent that women receive more SSRIs than men, whereas male AD recipients received more other ADs (e.g. Mirtazapine) compared to female AD users (SSRI: 49.7% male and 53.5% female; other ADs: 53.7% male and 49.8% female; *p* < 0.001). The level of urbanization did not vary meaningfully between the prescription rates in the drug classes (urban vs. rural area).

### Prescription provider

With regard to the source of prescription, data shows that 53.6% of the persons with ADs received their prescription by a GP only compared to 16.9% individuals receiving medication from a psychiatrist only (Table 5). The residual category “combination of providers” refers to all people who received two or more AD prescriptions by more than one different providers (16.3%) When considering the geographic area, in rural areas ADs are prescribed more often by GPs only compared to Psychiatrists only whereas the opposite is the case in urban areas (Table 5).

**Table 5:** Number of patients with class of AD prescriptions made by provider and area

	Provider^a^						
	GP N (%)	Psychiatrist N (%)	Other Specialist N (%)	Hospital ambulatory N (%)	Combination of providers N (%)	χ^2^ (df)	*P*-value
All ADs	56,669 (53.6)	17,832 (16.9)	6374 (6.0)	7589 (7.2)	17.199 (16.3)	782.00 (4)	< 0.001
Urban	43,302 (76.4)	15,261 (85.6)	5324 (83.5)	6069 (80.0)	13.694 (79.6)		
Rural	13,367 (23.6)	2571 (14.4)	1050 (16.5)	1520 (20.0)	3.505 (20.4)		
SSRIs	24,614 (23.3)	8230 (7.8)	2636 (2.5)	3296 (3.1)	16.460 (29.8)	389.73 (4)	< 0.001
Urban	18,875 (76.7)	7097 (86.2)	2222 (84.3)	2641 (80.1)	13.111 (79.7)		
Rural	5739 (23.3)	1133 (13.8)	414 (15.7)	655 (19.9)	3.349 (20.3)		
TCA	6130 (5.8)	1134 (1.1)	936 (0.9)	798 (0.8)	4.710 (34.4)	30.38 (4)	< 0.001
Urban	4727 (77.1)	946 (83.4)	768 (82.1)	629 (78.8)	3.674 (78.0)		
Rural	1403 (22.9)	188 (16.6)	168 (17.9)	169 (21.2)	1.036 (22.0)		
MAOIs	56 (0.1)	60 (0.1)	9 (0.001)	13 (0.001)	117 (45.9)	2.78 (4)	0.595
Urban	48 (81.4)	49 (81.7)	9 (100.0)	10 (76.9)	91 (78.1)		
Rural	11 (18.6)	11 (18.3)	0 (0.0)	3 (23.1)	26 (21.9)		
Others	22,586 (21.4)	7181 (6.8)	3002 (2.8)	4075 (3.9)	17.127 (31,7)	218.25 (4)	< 0.001
Urban	17,317 (76.7)	6026 (83.9)	2488 (82.9)	3255 (79.9)	13.580 (79.3)		
Rural	5269 (23.3)	1155 (16.1)	514 (17.1)	820 (20.1)	3547 (20.7)		

*χ*^*2*^ Chi square, *GP* General Practitioner; ^a^The provider type refers to patients with prescriptions made by this type of provider only; Chi-square-tests are related to the overall test of association between provider type and area of residence (urban vs. rural); See Methods section for further definitions of variables

## Discussion

The aim of the current study was to draw an accurate picture of the AD prescription prevalence and practices in the Swiss health care system based on data of a large health insurer. By using a health claims database that covers 1.17 million insurees, we identified 105,663 patients who were prescribed at least 1 AD medication in the year 2016. Overall, the rate of 8.7% for the use of any AD in Switzerland is slightly higher than the average of 7.2% detected in a large population survey in 27 European countries [18] and compared to the prescription prevalence of 7.4% in a German study using health claims data approach [19]. The most commonly prescribed AD classes were SSRIs and other ADs (mostly SNRIs), which were far more prevalent than TRCs and MAOIs. This pattern is not surprising and consistent with studies in other European countries, who demonstrate that the increase in AD prescription is largely driven by an increase in the dispensing of SSRIs and drugs in the other AD group [10, 7]. González-López et al. found that the rise in the latter group is primarily explained by increased dispensing of Trazodone, Venlafaxine, and Mirtazapine, the three most commonly prescribed drugs from the other AD group in our study.

The demographic characteristics of AD users broadly reflect previous findings that demonstrate a higher prevalence of AD use in women and older people (e.g., [19, 7, 10, 20]. Our analysis revealed that women are 50% more likely than men to receive ADs, and that prescription increases with age, posing a particular high likelihood of AD prescription in people ≥85 years old. While the present data and analysis does not allow to draw conclusions about the appropriateness of AD prescription as part of the treatment of a given depressive disorder, the embedding of our results in the context of depression prevalence is important to identify possible indications of inappropriate health care. In international epidemiological studies, the 12-month prevalence of depressive disorders is 7% [21]. In Switzerland, a population survey revealed a 12-month prevalence of major depression of 5.2% [12] which contrasts with the AD prescription rate of 8.7% in our study. While data has consistently shown that depression is more common in women, the Swiss study shows that women only have a 25% higher prevalence of depressive disorders compared to men. Yet, our analysis revealed that women are two times more likely to receive any AD compared to males. This finding applies to all classes of AD except for MAO inhibitors. One explanation for a higher AD prescription rate in women compared to men might be an overall higher rate of comorbid mental health issues in women, for which AD treatment is indicated, such as anxiety disorders, insomnia, and pain, while increased help-seeking in women compared to men might be another reason [22].

Our analysis showed that the likelihood of AD prescription rises with age and that the very old people have higher odds for AD prescription compared to younger adults. Due to our procedure of extrapolating age-related prevalences to the general population, we can rule out that under- or overrepresentation of certain age groups in our sample accounts for this age effect. There is supporting evidence that high age is a risk factor for AD prescription and use [23–25]. For example, in a study investigating time trends of AD prescription rates across lifetime, a rise in AD prescribing in all age groups, with a threefold increase of AD prescription in the older age group relative to the middle-aged group was observed [23]. This is notable in view of inconsistent results about the prevalence of depressive disorders in the elderly. Indeed, a recent epidemiological study in six European countries showed that affective disorders are highly prevalent in the older population: the 12-month prevalence of affective disorders in people aged 65 to 84 years was 13.7% [26]. However, it has also been argued that major depressive disorder is relatively rare amongst old people but that minor depression explains the prevalence of depression in older age [27]. A finding from a Swiss population health survey demonstrates that the perceived depression decreases as people get older, indicating that elderly people experience and report less depressive symptomatology [13]. Additionally, evidence for high rates of AD prescription in older populations despite absence of a diagnosis of a major depressive disorder or significant depressive symptoms [25], points toward potential overuse of AD in the very old. Finally, our finding of relatively high AD prescription rate of 16.2% in individuals aged ≥65 is in contrast with a recent Swiss survey according to which only 4.7% of the people above 65 years received ADs [13]. While multiple factors might influence high AD prescription in the old population, lacking alternatives of psychosocial and psychotherapeutic care might partially contribute to this finding.

Higher number of comorbid somatic conditions increased the likelihood of AD use by up to 13 times. Although this result might reflect the growing body of evidence demonstrating the high rate of somatic comorbidities in depression [28], it should further be investigated, whether increasing prescription rates of AD for non-depressive and off-label indications might be one factor contributing to that result [29].

Differences in the regional distributions of AD prescription rates are another important finding that warrants further exploration. There are substantial differences in prescription rates on a canton level, ranging from 6.5 to 11.9% when adjusting for regional distribution of the Swiss population. This might be due to different health services structures in these regions, e.g. variations in physician density. The comparison of our prevalence rates with statistics on physician density per inhabitant and the net benefit costs per insured persons in the year 2016, the following picture emerges: There is a clear congruence of low prescription rates in cantons with a low density of medical doctors and a similar but not conclusive relationship of high AD prescription rates in cantons with high density of medical doctors [30, 17]. This pattern remains stable for all cantons with low prescription rates and most cantons with high prescription rates after extrapolating to the general Swiss population. This finding is in line with a systematic review, which identified a consistent significant association between physician density and health care consumption [31]. A German study examining regional differences in AD prescription based on health claims data also argued that low prescription rates in some regions might be due to a lack of health care professionals [19].

Lastly, we examined the characteristics of the prescription patterns as a function of the prescription source or health care provider. We found that the majority of patients was prescribed the AD medication exclusively by a GP. Just a small fraction of ADs was prescribed by psychiatrists only, physicians only, who work in hospital ambulatories, and by other specialists. GPs and outpatient psychiatric practices are usually the first point of contact for patients with mental complaints and provide the major part of outpatient care for individuals with mental disorders. There is evidence that in primary care depression and mixed anxiety disorders account for 60 to 75% of AD prescriptions [32, 33]. Our finding thus might reflect a generally low referral rate of patients with depression to specialized care – in Switzerland only 13% [13]. An additional finding paralleling this interpretation is the higher AD prevalence found in individuals registered with standard care plans compared to insurees registered with Managed Care. From a health care system perspective managed care models are associated with higher referral rates in Switzerland [34]. While two thirds of our sample were enrolled in Managed Care, this only applies to a vastly smaller fraction of the Swiss general population [35]. Taken together, these important observations prompt questions of whether the intersection between primary and secondary care bears challenges in the Swiss health care system, which lead to a high load of and potentially inappropriate AD treatment delivered in primary care.

### Strengths and limitations

We would like to point out three major weaknesses of our study design and methodology. Firstly, as we are examining AD prescription rates based on health claims data, we cannot be sure that persons, who receive an AD prescription take this medication as prescribed. Since insurance claims data require a patient to pick up the prescribed drug in a pharmacy, it would be rather unlikely for an individual to obtain the prescribed medication without consuming it. Secondly, it is important to mention that the data of this study are not entirely representative of the Swiss general population, with minor differences being observed regarding sex, age and the region of residence. In order to correct for potential biases, we provided raw and adjusted results. However, several strengths of the data need to be stressed as well: The results presented in the study were based on a population of over 1 million insured subjects from all regions of Switzerland; the obligatory health insurance provided is defined at the federal level and it is the same for all health insurance companies; the data quality is excellent due to a high degree of completeness. For these reasons a high degree of generalizability of the data can be assumed.

Lastly, and most importantly, this study lacks any diagnostic information about the people who receive AD. This makes conclusions of the (in-) appropriateness of AD treatment difficult. Thus, the data illustrates the prevalence, patterns, amount and source of AD prescriptions in a collective of insured patients but it does not necessarily relate to AD prescription in diagnosed patients with depression. Yet, we believe that AD prescription rates serve as a proxy for the treatment of depression and depressive symptoms allowing for comparisons of depression prevalence rates and AD prescription rates which might shed light on the appropriateness of mental health services in Switzerland.

This is important given the lack of complete and appropriate data with regard to psychotropic drug dispensing in the Swiss health care system. For example, there is one data source in Switzerland indicating that more than two thirds of patients with less severe forms of depression are treated exclusively with ADs in primary care [12]. This issue is of particular interest given that the proportion of patients expressing a preference of psychological treatment is three times higher than those preferring pharmacological treatment [36]. Furthermore, this “real-world information” analysis adds objective and reliable data to previous work which was mainly based on self-report information. Especially in the context of stigma related to mental disorders, it is important to have access to unbiased data, which are not influenced by social desirability, a lack of reliability and other sources of bias related to self-reporting.

## Conclusions

Overall, our analyses showed that AD prescriptions are more prevalent than expected, particularly in older age groups, and are subject to regional variation. In Switzerland, AD treatment seems to be primarily delivered by GPs. Taken together these results call for further, more detailed analyses in this important field. Results from routine data are essential since it provides a basis and starting point for further investigation of prescription practise. A continued effort to build on these data would enable researchers and clinicians to track timing of current AD treatments as well as time trends of AD treatment in the Swiss health care system. AD is primarily used for the treatment of depressive disorders and the pharmacological treatment of depression is subject to specific recommendations, guidelines and principles [4]. Further investigation of AD prescription patterns, such as duration, combination, switching, and augmentation, as well as treatment trajectories of AD prescription will increase our understanding of the pharmacological treatment practices of mental disorders and particularly depressive disorders. The current analysis can be an important first step to carry forward a meticulous investigation of routine data regarding AD prescription in Switzerland.

## Additional files

Additional file 1: **Figure S1.** Raw and adjusted AD prescription rates by age group (in years). (DOCX 1803 kb)

Additional file 2: **Figure S2.** Raw (black) and adjusted (red) AD-prescription rates by cantons. (DOCX 2545 kb)

## Data Availability

The data that support the findings of this study are available from Helsana but restrictions apply to the availability of these data, which were used under license for the current study. Data are thus not publicly available. Data are however available from the authors upon reasonable request and with permission of Helsana.

## Abbreviations

AD: Antidepressants
ATC: Anatomical Therapeutic Chemical
CCC: Comorbid chronic condition
GP: General Practitioner
MAOIs: Monoamine oxidase inhibitors
SHS: Swiss Health Survey
SNRIs: Serotonin-norepinephrine reuptake inhibitors
SSRIs: Selective serotonin reuptake inhibitors
TCA: Tricyclic antidepressants
