# Supplementary material for: Antidepressant prescription practice and related factors in Switzerland: a cross-sectional analysis of health claims data
Source: BMC Psychiatry. 2019 Jun 24;19:196. doi: 10.1186/s12888-019-2178-4 (PMC6591836; doi:10.1186/s12888-019-2178-4)
Supplement: Supplementary file 1 — Figure S1. Raw and adjusted AD prescription rates by age group (in years). (DOCX 1803 kb) [file 12888_2019_2178_MOESM1_ESM.docx]

Additional file 1
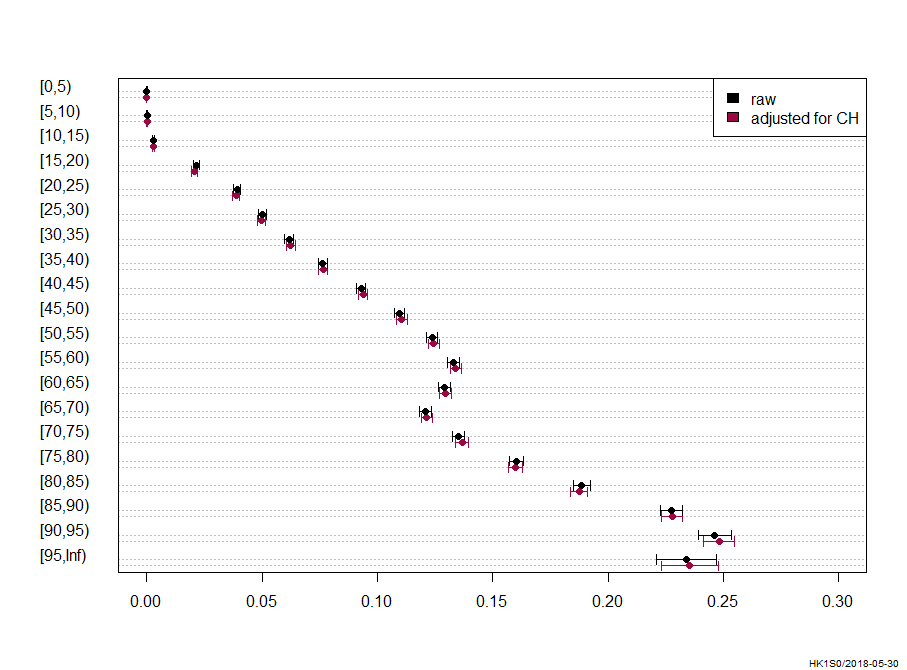
Figure S1: Raw and adjusted AD prescription rates by age group (in years). **Note.** Parenthesis includes threshold value, bracket does not include threshold value.
