# Supplementary figures and images for: Antidepressant prescription practice and related factors in Switzerland: a cross-sectional analysis of health claims data
Source: BMC Psychiatry. 2019 Jun 24;19:196. doi: 10.1186/s12888-019-2178-4 (PMC6591836; doi:10.1186/s12888-019-2178-4)

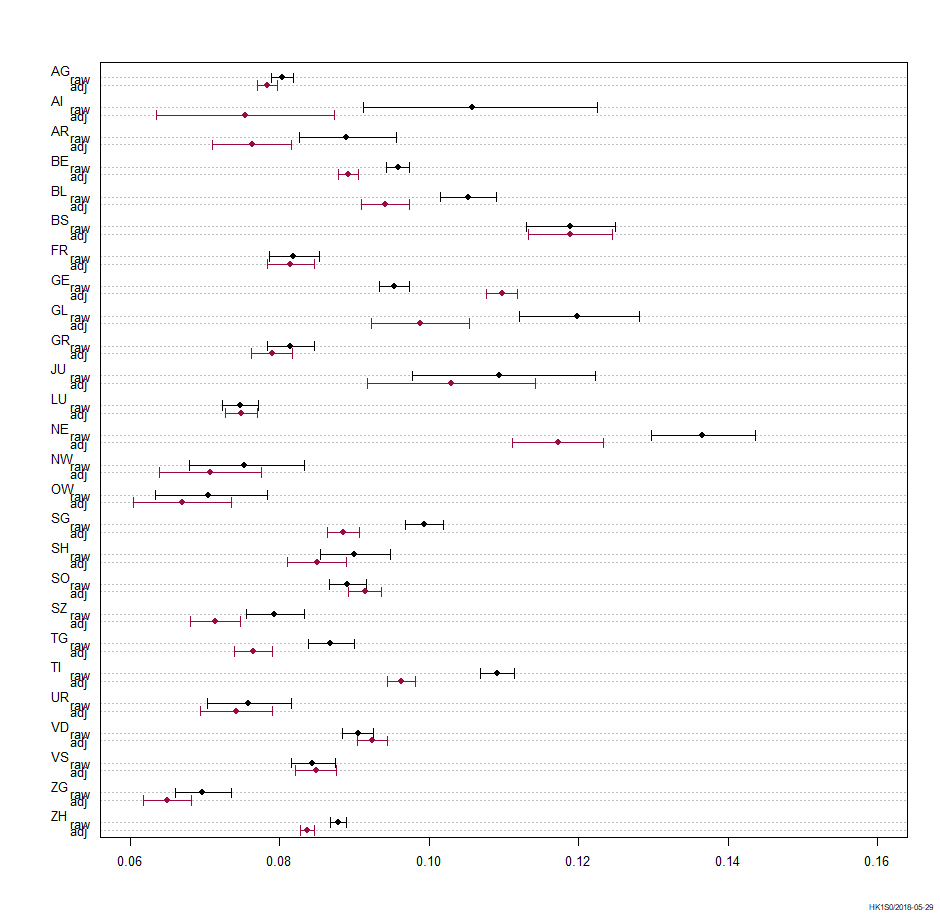


Figure S2: Raw (black) and adjusted (red) AD-prescription rates by cantons.

Supplement: Supplementary file 2 — Figure S2. Raw (black) and adjusted (red) AD-prescription rates by cantons. (DOCX 2545 kb) [file 12888_2019_2178_MOESM2_ESM.docx]
